# Spin Selectivity Damage Dependence of Adsorption of dsDNA on Ferromagnets

**DOI:** 10.1021/acs.jpcb.2c08820

**Published:** 2023-03-08

**Authors:** Kakali Santra, Yiyang Lu, David H. Waldeck, Ron Naaman

**Affiliations:** † Department of Chemical and Biological Physics, Weizmann Institute, Rehovot 76100, Israel; ‡ Chemistry Department, University of Pittsburgh, Pittsburgh, Pennsylvania 15260, United States

## Abstract

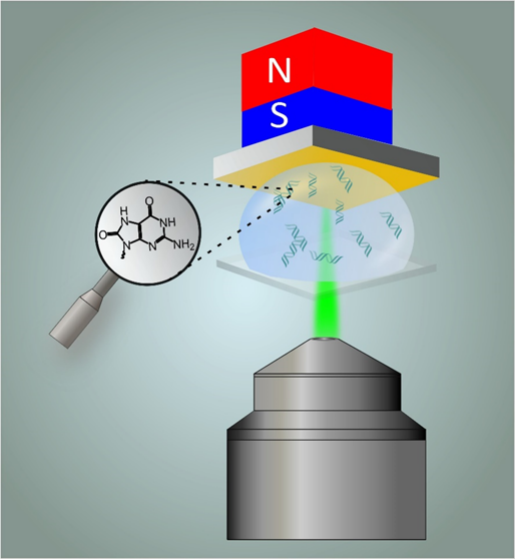

The adsorption of oxidatively damaged DNA onto ferromagnetic substrates was investigated. Both confocal fluorescence microscopy and quartz crystal microbalance methods show that the adsorption rate and the coverage depend on the magnetization direction of the substrate and the position of the damage site on the DNA relative to the substrate. SQUID magnetometry measurements show that the subsequent magnetic susceptibility of the DNA-coated ferromagnetic film depends on the direction of the magnetic field that was applied to the ferromagnetic film as the molecules were adsorbed. This study reveals that (i) the spin and charge polarization in DNA molecules is changed significantly by oxidative damage in the G bases and (ii) the rate of adsorption on a ferromagnet, as a function of the direction of the magnetic dipole of the surface, can be used as an assay to detect oxidative damage in the DNA.

## Introduction

The central dogma of biology holds that the nucleobase sequence of DNA carries the specific genetic information that is translated to RNA and proteins, which manifests in an organism’s phenotype. While sequence preservation is important, some amount of mutation and/or damage is essential for natural selection and evolution. The base pairs are susceptible to damage arising from cellular respiration, environmental exposure to free radicals, and other factors.^1^ An example of such damage is the 7,8-dihydro-8-oxoguanine (OG), or other oxidized guanine products, which results commonly because of the guanine base pair’s low reduction potential. Understanding the cellular mechanism for detecting and repairing such damage, to inhibit mutagenesis, is of great interest.^2^ Because OG has a minor effect on the structure and stability of DNA, the mechanism through which repair enzymes locate it remains unknown.

We hypothesize that spin polarization differences can be used to distinguish OG DNA from its undamaged form. This hypothesis is motivated by three different observations. First, studies have demonstrated that OG damage improves spin-selective electron transmission through DNA.^3^ Second, it has been shown that spin polarization in chiral biomolecules (nucleic acids, peptides, and amino acids) affects their interaction with ferromagnetic surfaces.^4,5^ Third, the interaction between oligopeptides and double-stranded DNA (dsDNA) molecules, adsorbed on ferromagnetic surfaces, is spin-dependent.^6^ To test this hypothesis, we investigated the adsorption of a double-stranded DNA on ferromagnetic (FM) films and compared it to the case of OG-damaged DNA.

We examined the adsorption of four DNA duplexes on Ni/Au surfaces: one is an unmodified DNA duplex and the other three are different OG-damaged DNA duplexes on FM surfaces. In these experiments, the damaged DNA duplexes differ by the location of an OG damage site, which was systematically varied along the duplex DNA’s helix; the undamaged DNA duplex serves as a control system. The FM substrate, a Ni/Au film, was magnetized perpendicular to the substrate plane, and the adsorption was monitored in two different ways, confocal fluorescence microscopy and quartz crystal microbalance (QCM) measurements. Previously, we used QCM to show that the adsorption kinetics of a chiral amino acid, cysteine, on an FM surface is enantiospecific with the FM electrode’s magnetization direction along the surface normal.^7^ In this work, confocal laser scanning microscopy (CLSM) and QCM studies show that the adsorption rate and the total coverage of DNA on ferromagnetic substrates depend on both the magnetization direction of the FM and the presence of OG damage, as well as its position in the DNA duplex. These findings are rationalized by considerations arising from the chiral-induced spin selectivity (CISS) effect.^8^

## Experimental Methods

### Ferromagnetic Substrate Preparation

The ferromagnetic substrates were prepared by the deposition of a 100 nm thick Ni layer on a p-type (Boron doped) Si ⟨100⟩ ± 0.9° wafer in an e-beam evaporator. An 8 nm Ti layer was coated between the Si and Ni as the adhesive layer. The nickel layer was coated with a 5 nm thin layer of Au. Previous reports described the fabrication of the surface and its use in CISS effect applications.^9,10^ The chamber was maintained at a high vacuum (<10^–7^ torr) and at ambient temperature during the deposition of the metallic layers. For the quartz crystal microbalance (QCM) measurements, 10 nm of Au was coated on the 100 nm Ni layer. The substrates were diced into 23 × 23 mm^2^ sized squares for all of the confocal experiments. Before evaporation, the pieces of substrates were cleaned by boiling in acetone and ethanol, each for 10 min.

### DNA Hybridization

DNA with the fully matched sequence and all of the complementary strands tagged with cyanine3 (cy3) dye were purchased from Integrated DNA Technologies (IDT Synthezza, HPLC purified with mass spectroscopy certificate of analysis). The cy3 dye was covalently attached to the 3′ end of the complementary strand. We performed the DNA hybridization as reported elsewhere.^11^Figure S3 shows the CD spectra and the corresponding UV–vis spectra of all of the double-stranded DNA molecules. The concentration was determined from the signature absorption intensity of the double helix DNA at 260 nm using a Thermo Scientific Nanodrop ONE^C^ UV–vis spectrometer. Then, it was diluted with 0.4 M phosphate buffer (pH 7.2) to a final concentration of 0.5 μM. CD spectroscopy was used to confirm the hybridization of the DNA molecules.

The sequences of the base pairs (5′ → 3′) are as follows:

40nt dsDNA with the fully matched sequence: “Full-match’’

(1) 5′-TAT ATA TTA TTC TTA TTA TTA TTC TTT ATA TTT TTT TTT T-(CH_2_)3-S–S-(CH2)_3_-OH
(2) 3′-Cy3-ATA TAT AAT AAG AAT AAT AAG AAA TAT AAA AAA AAA A-5′

In the case of the OG damage, the following sequences were used, where **8** = 8-oxo-dG: 40 nucleotide (40nt) dsDNA with distal oxidative damage in guanine: “Distal OG”

(1) 5′-TAT ATA TTA TTT TTA TTA TTA TTT TTT AT**8** TTT TTT TTT T-(CH2)_3_-SS-(CH_2_)_3_-OH
(2) 3′-cy3-ATA TAT AAT AAA AAT AAT AAT AAA AAA TA**C** AAA AAA AAA A-5′

40nt dsDNA with central oxidative damage in guanine: “Central OG”

(1) 5′-TAT ATA TTA TTT TTA TT**8** TTA TTT TTT ATA TTT TTT TTT T-(CH2)_3_-SS-(CH_2_)_3_-OH
(2) 3′-cy3-ATA TAT AAT AAA AAT AA**C** AAT AAA AAA TAT AAA AAA AAA A-5′

40nt dsDNA with proximal oxidative damage in guanine: “Proximal OG”

(1) 5′-TAT AT**8** TTA TTT TTA TTA TTA TTT TTT ATA TTT TTT TTT T-(CH2)_3_-SS-(CH_2_)_3_-OH
(2) 3′-cy3-ATA TA**C** AAT AAA AAT AAT AAT AAA AAA TAT AAA AAA AAA A-5′

The site-specific oxygen-damaged DNA strands were obtained from Prof. Cynthia J. Burrows, Department of Chemistry, Utah University, and were prepared as described in reference (6).

### Adsorption Kinetics of DNA on Gold-Coated Ferromagnetic Substrates

To verify the importance of the electrons’ spin in the adsorption of the DNA molecules, we measured the dependence of the rate of adsorption on the ferromagnetic (FM) substrate when the FM layer was magnetized perpendicular to the surface, directed either away from the surface (Up) or into the surface (Down) by using a permanent 0.42 T magnet. The adsorption occurred through a strong covalent Au–S bond between the gold layer and the thiol-functionalized DNA molecules. All of the OG DNA strands, used in this work, consisted of an identical sequence of base pairs differing only at the location of the OG.

We carried out the adsorption of the molecules using a 0.5 μM solution of the dsDNA in 0.4 M phosphate buffer (pH = 7.2). A 110 μL aliquot of the DNA solution was drop-cast at the center of the MAKTEK glass-bottom-well Petri dish, which was then placed on the microscope stage. The magnet was placed precisely above the ferromagnetic substrate, with the ferromagnetic layer side facing the dsDNA-containing buffer solution. The timer was instantly set, and the images were collected at different time intervals of up to 20 min, with the surface magnetized with the North pole of the permanent magnet either Up (north) or Down (south).

### Microscope Setup and Data Analysis

We performed the fluorescence imaging experiments using a ZEISS LSM 800 confocal laser scanning microscope aligned in an inverted fashion. For the current experiments, we used a 561 nm (10 mW) diode laser. The laser beam was focused using a 10× objective lens (EC Plan-Neofluar, N. A. 0.3). We illuminated the sample with 1.5% of the laser power. The emitted fluorescence was collected by the same objective lens and was separated from the excitation beam by placing two dichroic beam splitters in the optical path. It was then routed to an avalanche photodiode (APD) for fluorescence imaging. Before entering the APD, the luminescence beam passed through a narrow pinhole that blocked all of the stray light or fluorescence that comes from the out-of-focus planes of the substrate–solution specimen. The emission was collected from 571 to 700 nm. Here, we focused the *z*-plane at the surface–solution interface and it was fixed for all of the measurements. The *x*–*y* coordinate of the stage was also fixed.

The snaps were acquired using ZEN 2.3 and processed with ImageJ and then in Origin software. We selected the same region from each image with a size of 400 pixels × 400 pixels. The mean fluorescence intensity values were analyzed using identical operations. Each experiment was repeated three times to ensure the reproducibility of the results. The errors shown in the plots were calculated as the standard deviation from the mean.

### Open Circuit and Contact Potential Difference Measurements in Quartz Crystal Microbalance

The open circuit and the contact potential difference experiments were performed using a 7.9995 MHz quartz crystal with an EQCM cell attachment and a 430A potentiostat (CH Instruments). The surface area of the crystal was 0.205 cm^2^ and was coated with 100 nm of nickel and 10 nm of polycrystalline gold as the working electrode. The counter electrode was a Pt wire, and the reference electrode was Ag/AgCl (saturated). The sample was first scanned from −0.4 to – 0.9 V, versus saturated Ag/AgCl, at a scan rate of 25 mV/s for 10 cycles to allow the electrochemical setup to equilibrate and give consistent results (shown in Figure S2). Then, the potential was held at −0.9 V for 1 min to fully desorb the DNA molecules and switched to an open circuit condition for the adsorption of DNA. The mass change was collected immediately after switching to the open circuit condition. Each sample was repeated 6 times.

For contact potential difference measurements, the electrode was first incubated in the 0.5 μM fully matched DNA solution for 30 min to allow for DNA adsorption on the surface and to allow for system equilibration. Then, the sample was scanned from 0 to −1 V versus saturated Ag/AgCl at a scan rate of 25 mV/s for 3 cycles and the potential where the current crossed zero was collected on the 3^rd^ cycle. Each sample was collected in the order of no magnet, North, and South. Four 0.5 μM fully matched DNA solutions were measured for each duplex type.

### X-ray Photoelectron Spectroscopy (XPS)

The adsorption of full-match dsDNA molecules on Ni/Au substrates was analyzed by XPS measurements using a Kratos Axis Ultra DLD spectrometer equipped with a monochromatic Al Kα X-ray source (*h*ν = 1486.6 eV) operating at 75 W. Measurements were performed at a 0° emission angle with respect to the surface normal. Elemental concentrations of S 2p and N 1s were measured from the relative intensities of the surface-adsorbed dsDNA molecules.

### Superconducting Quantum Interference Device (SQUID) Measurements

Magnetic measurements of the (Ni/Au) layer were performed using an MPMS3 SQUID magnetometer (LOT-Quantum Design Inc.). The ferromagnetic layer (Ti 8, Ni 100, Au 5 nm) was coated on a 4 mm × 4 mm sized Si substrate. A magnetic field of up to 6 T was applied out-of-plane to the substrate. We measured the adsorption of full-match DNA and the central OG DNA on the Ni/Au substrate magnetized with the external magnet pointing to the North or South pole for up to 20 min.

## Results

**Figure 1:**
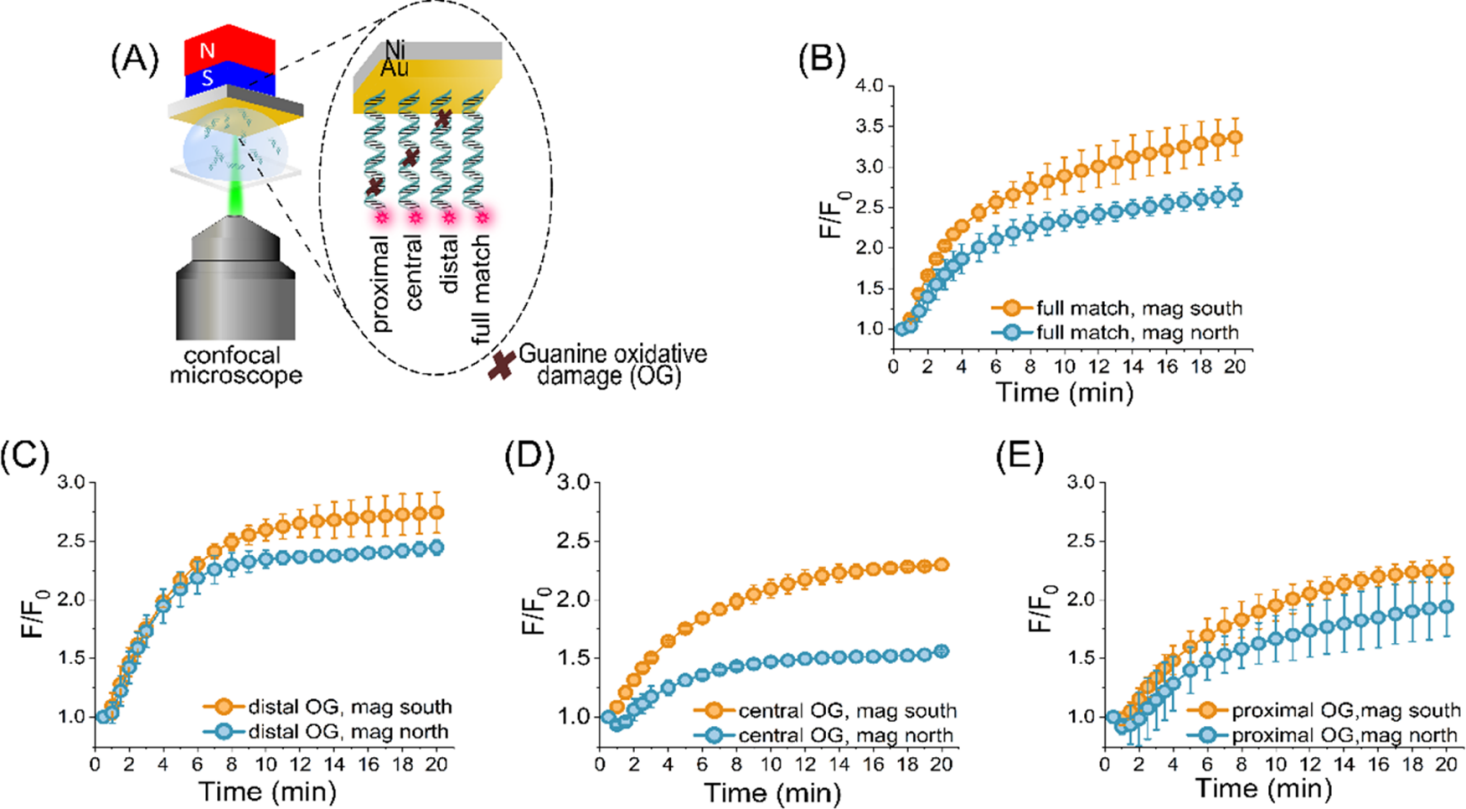
Results for the spin-dependent adsorption of the double helix DNA molecules on a Ni/Au-coated ferromagnetic (FM) film by confocal microscopy. (A) Schematic illustration of the DNA-cy3 molecules assembled on the FM substrate. The cross in the schematic marks the location of OG in the duplex strand, and the star indicates the location of the attached dye on the free end of the duplex. The adsorption is performed for both north and south orientations of the spins in the ferromagnetic substrate aligned by the external magnet beneath the substrate. (B–E) The fluorescence intensities are observed from the magnetized ferromagnetic surface. The normalized *F*/*F*_0_ values are shown as a function of adsorption time, where *F* is the fluorescence intensity measured and *F*_0_ is the background signal measured prior to the adsorption of the dsDNA strands. Results are shown for the fully matched and the three 8-oxo-dG DNA duplexes: distal, central, and proximal positions. The excitation wavelength for the cyanine is 561 nm, and the emission is collected from 571 to 700 nm.

**Figure 2:**
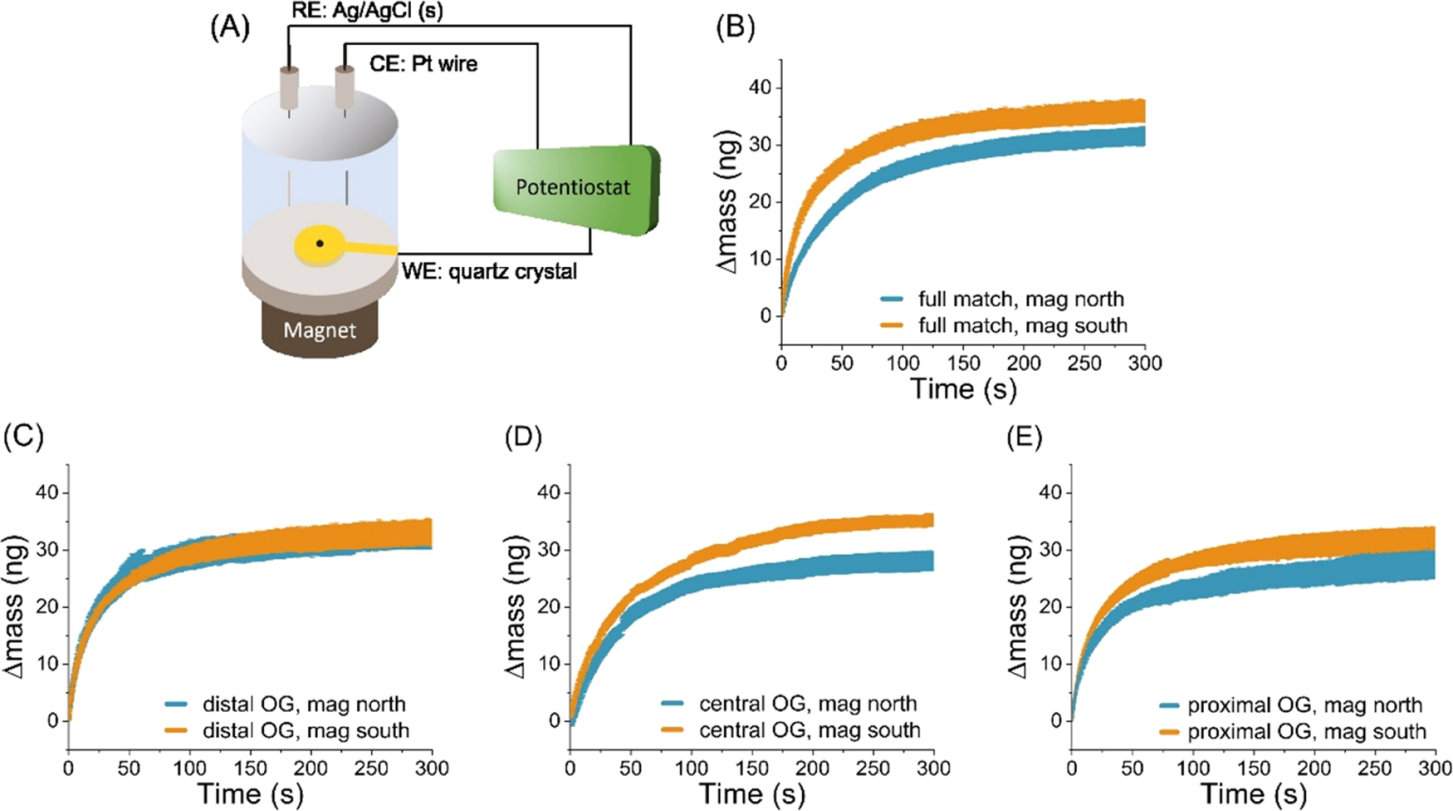
Results for the spin-dependent adsorption of the double helix DNA molecules on a Ni/Au-coated ferromagnetic (FM) film by quartz crystal microbalance. (A) Schematic showing the electrochemical quartz crystal microbalance system, in which DNA duplexes adsorb onto a magnetized Ni/Au film electrode. The mass change of the adsorption was monitored under open circuit conditions to mimic the fluorescence experiments. (B–E) Mass change measured for north magnetic field (blue) and a south magnetic field (orange) for 0.5 μM full-match DNA and distal, central, and proximal OG DNA solution, respectively.

The real-time adsorption of duplex DNA on an FM substrate was followed by monitoring the time-dependent fluorescence from cy3 dye-tagged dsDNA molecules. The substrate was silicon-coated with a Ti/Ni/Au (8/100/5 nm) film, and it was magnetized with the North pole of the magnetization pointing either toward or away from the adsorbed layer. A permanent magnet was used for magnetizing the Ni/Au film, and the magnet was maintained in proximity to the surface for the duration of the experiments (see Figures 1A and 2A) with its North or South Poles pointing toward the surface. The DNA was adsorbed from a 0.5 μM solution of the dsDNA in 0.4 M phosphate buffer (pH ∼ 7.2). The dsDNA molecules contained OG damage at three different locations, namely, as distal, central, and proximal with respect to the cy3 at the 3′ end on the secondary strand (see Figure 1A and ref^6^).

The fluorescence (F) was monitored for up to 20 min, starting when the substrate was dipped into the solution containing the DNA in a bottom-well Petri dish. The signal is normalized to the background fluorescence, F_0_, prior to any significant amount of DNA adsorption on the surface. The time was measured from the insertion of the ferromagnetic film-coated substrate to the solution. It was found that the adsorption of all of the DNA duplexes was higher for the South pole of the magnetic field pointing toward the solution than for the North pole oriented toward the solution and that the asymptotic value of the fluorescence intensity for the fully matched, undamaged DNA (Figure 1B) was higher than that of the damaged DNAs (Figure 1C–E). The observed difference in the fluorescence intensity as a function of the direction of the magnetic field indicates a spin-dependent interaction. In contrast to the earlier work,^8^ which examined the enantiospecific interaction between chiral molecules and an FM substrate for a short time, the studies reported here are extended to long times where the coverage approaches an asymptotic limit.

The difference in the coverage of adsorbed DNA molecules (amount), as a function of the substrate’s magnetization direction, was quantified by X-ray photoelectron spectroscopy (XPS). The elemental peaks of N 1s and S 2p confirmed that the DNA molecules were chemisorbed on the surface for both directions of the magnetic field. The limited signal-to-noise ratio caused the elemental S 2p signal at 162 eV to be inconclusive. The changes in the atomic percentage from the elemental N 1s signal at a 401 eV binding energy (Figure S1) showed a very small difference in the intensity, however. We normalized the intensities of each peak by the Au 4f signal, and for each sample, we measured the intensity at two random points on the surface. Table S1 shows the elemental atomic percentages and normalized percentages that were obtained by this process.

Figure 1C–E shows the time-dependent chemisorption for distal, central, and proximal OG-damaged DNAs. The adsorption curves reach different asymptotic intensities based on the location of the damage in the helix. The intensity decreases from distal to central under south magnetization; however, any change from central to proximal OG is not evident. Similar to the undamaged DNA, the difference in the adsorption as a function of the direction of the magnetic field indicates spin selectivity for the adsorption. The effect is larger in the case of the central OG DNA and is the smallest for the distal and proximal OG.

The findings from the fluorescence studies are corroborated by quartz crystal microbalance (QCM) studies. Figure 2A shows a schematic diagram for the electrochemical QCM measurement, which reports the mass of the adsorbed molecules as a function of time. Note that the time scale of the signal is somewhat different from that in Figure 1 because the geometry of the adsorption cell is different. Namely, here the surface, on which the adsorption was measured, was larger than in the case of the fluorescence studies (0.205 vs 0.002 cm^2^, respectively), while the volumes of the solution were 2 mL and 110 μL in the QCM and fluorescence studies, respectively. These differences arise from constraints of the experimental apparatuses.

As shown by Figure 2 and Table 1, the mass changes for the adsorption of all four different DNAs are around 30–40 ng within 300 s. As in the case of the fluorescence studies, the adsorption rate and the amount of molecules adsorbed on the surface are higher when the magnetic South pole is pointing toward the molecules. In addition, the differences in the coverage between the North and South pole orientations follow the same trend as in the fluorescence studies. The correlation between the fluorescence and the QCM data indicates that indeed the adsorption depends on the direction of the magnetic field acting on the FM substrate and on the position of the damage in the DNA. In contrast to earlier studies, which found that the rate of chiral molecule adsorption changes on FM substrates but its total coverage does not,^8^ we find that both the rate of adsorption and the total coverage of DNA depend on the magnetization of the FM electrodes.

**Table 1:** Mass Change in 300 s, in Units of ng, and the Corresponding Coverage, in Units of 10^12^ molecules/cm^2^, on the Ni/Au Films Is Reported for Different Positions of the Damage on the DNA^a^

	**South**		**North**	
	mass change	sample coverage	mass change	sample coverage
fully matched	36.07 ± 1.62	4.20 ± 0.18	31.50 ± 1.30	3.67 ± 0.15
distal OG	33.18 ± 1.95	3.87 ± 0.22	31.91 ± 1.66	3.72 ± 0.19
central OG	35.35 ± 0.88	4.12 ± 0.10	28.20 ± 1.61	3.29 ± 0.19
proximal OG	31.94 ± 1.90	3.72 ± 0.22	27.17 ± 2.10	3.17 ± 0.24

a South and North indicate the direction of magnetic pole pointing toward the solution.

When comparing the ratio between the signals observed for the South- and North-magnetized FM films (see Table 2), the two methods provide the same trends; namely, the largest ratio is obtained for the central OG, while the smallest ratio is obtained for the distal OG. However, the ratios are consistently larger for the fluorescence as compared to the QCM studies. A plausible explanation for this difference is a more efficient quenching of the fluorescence from the dye when the North magnetic pole is pointing toward the adsorbed molecules. The spin-dependent electron transfer for the quenching of fluorescence for chiral assemblies^12,13^ and the spin-dependent photocurrent from a dye chromophore through a chiral bridge on electrodes^14^ has been reported before. This behavior can be rationalized by an electron-transfer-mediated quenching mechanism, in which the DNA preferentially transmits one electron spin direction into the FM over the other, which is affected by the direction of the magnetic dipole of the FM. Hence, spin-dependent fluorescence quenching by electron transfer to the substrate supports the increase in contrast for the adsorption differences with the magnetization direction that are measured by fluorescence, as compared to those measured by the mass changes.

**Table 2:** Ratio of the Asymptotic Signal Obtained When Adsorption Is Performed with either the South or North Magnetic Poles Pointing toward the Solution for the Fluorescence (F) and for the Quartz Crystal Microbalance (M) Studies^a^

sample	fluorescence ratio, F	QCM mass ratio, M	F/M
	South/North	South/North	
full match	1.27 ± 0.11	1.14 ± 0.07	1.11 ± 0.12
distal OG	1.12 ± 0.10	1.04 ± 0.08	1.08 ± 0.13
central OG	1.47 ± 0.06	1.25 ± 0.08	1.18 ± 0.09
proximal OG	1.16 ± 0.16	1.18 ± 0.11	0.98 ± 0.17

a The last column shows the ratio between the signals obtained by the two methods.

Note that the observed differences in adsorption rates, as a function of the magnetization direction of the substrates, cannot stem from magnetic force acting on the diamagnetic DNA because the observations here depend on the sign of the magnetic field and the Kelvin force does not depend on the sign of the magnetic field.

## Discussion

The duplex DNAs that were studied have identical base pair sequences and differ only in the presence and/or location of the OG defect. Thus, the difference in the intensity and the dependence on the magnetic field must originate from the difference in the location of the OG. Because the DNA duplexes are chiral, they should become charge and spin-polarized as they approach and bind to the metal surface, according to the CISS effect.^15^ Because of the transient spin polarization in the molecule, a spin exchange interaction manifests between the thiol group on the DNA and the magnetized FM substrate, leading to DNA chemisorption, which depends on the FM substrate’s magnetization, i.e., spin alignment. Namely, the adsorption will be faster for the case where most of the spins in the Ni layer are aligned antiparallel to the polarized spin at the molecule’s binding site, and the difference in the rate of adsorption should correlate with the extent of charge and spin polarization in the DNA.^8^

It is interesting that the preferred magnetic field direction, as observed in the current studies, is opposite to that obtained in reference (8). To verify the reason for it, we repeated the experiment with the same DNA sequence used in ref (8) and found that the preferred magnetic orientation is South, as observed in the present study (see Figure S4). In ref (8), the adsorption was controlled by the kinetics and it was performed under different conditions as in the present study. Here, we worked at much lower concentrations and followed the adsorption in situ. Hence, we obtained the thermodynamic equilibrium, as discussed below. We propose that this is the reason for the discrepancy in the results. However, this subject of kinetics vs thermodynamics is the focus of future studies.

**Figure 3:**
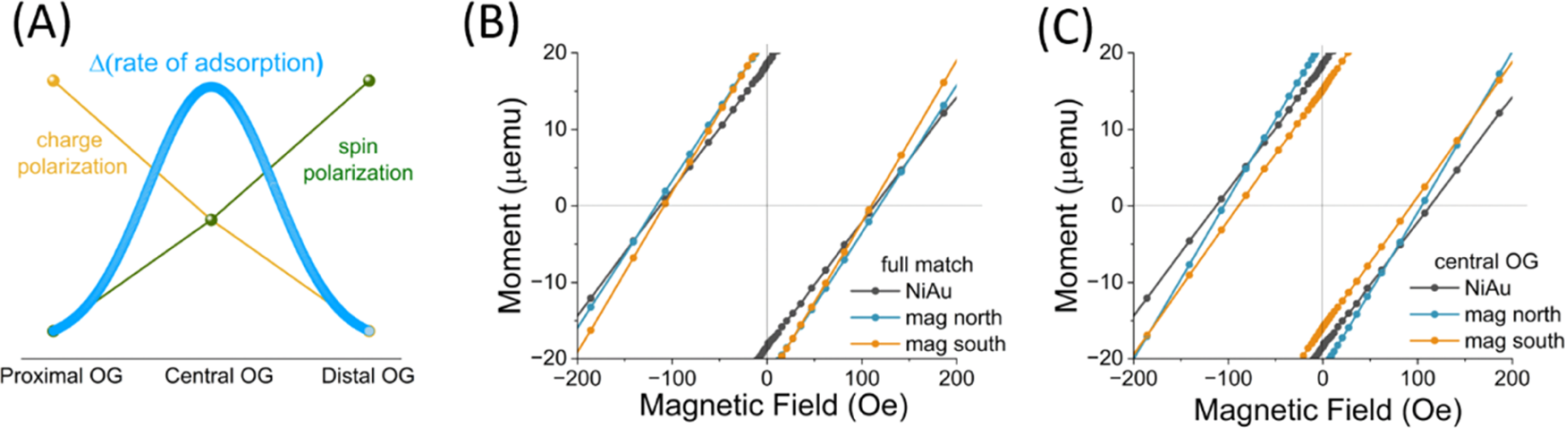
(A) Schematic of the model showing how the combination of charge and spin polarization affects the rate of OG DNA adsorption on a ferromagnetic substrate. (B) SQUID results of the full-match DNA adsorbed on a Ni/Au substrate when the substrate was magnetized with the magnet either North or South pole up direction. The bare Ni/Au substrate was measured as a reference. (C) SQUID results of the central OG DNA adsorbed on Ni/Au when the substrate was magnetized with either the North or South pole direction of the magnet. Measurements were conducted at 300 K.

The damage in the DNA inhibits the charge polarization; however, it enhances the spin polarization of the electrons that succeed to pass through it.^16^ Thus, the trend in the observations with the OG defect position results from the combination of two counteracting effects. The charge polarization is the largest for the damage being farthest from the substrate and is the smallest for the damage being closest to it; see Figure 3A. In contrast, the spin polarization is smallest when the damage is farthest from the substrate and highest when the damage is located closest to the surface.^16^ We posit that the difference in the selectivity of the adsorption rate, Δ*R*, is given by Δ*R* = *k*·*S*·*C*, where *k* is a proportionality constant, *C* is the amount of charge polarization, and  is the spin polarization, with *N*_+_ and *N*_–_ being the amount of electrons polarized with spin parallel to the direction of polarization and/or opposite to it. As shown schematically in Figure 3A, the maximum of the differential adsorption rate is obtained when the product of the charge polarization and the spin polarization is the largest, which is expected for the case of the OG defect located near the center of the duplex.

In addition to the differences in the adsorption rates, the final coverage of the adsorbed molecules changes with the OG damage location and the magnetization direction of the FM substrate. If the effect of the spin-selective adsorption was purely a kinetic effect, then one would expect that the adsorption would result in the same coverage for all cases at long enough times. The difference in total coverage implies that the Gibbs energy for adsorption changes for the two magnetization directions. To probe the possible reason for the differences in coverage, we used a SQUID magnetometer to measure the magnetic coercivity of the substrates after the adsorption experiments in solution were completed. Figure 3B,C shows the magnetic moment as a function of the magnetic field for surfaces prepared when the substrate was magnetized in one way or the other. For both the full-match and central OG DNAs, the magnetic coercivity is larger for the case in which a North-magnetized FM layer was used to adsorb the DNA, i.e., lower coverage condition, and is smaller for the South magnetization condition.

For the central OG DNA, the magnetic moment increases more sharply with the applied magnetic field and reaches a somewhat larger saturation magnetization in the case when the film was prepared with a North-magnetized FM layer, i.e., lower coverage (shown in Figure S6B) than that of the South-magnetized layer. In additon, a decrease in the magnetic moment is observed when further increasing the applied field, indicating a diamagnetic contribution. The magnetometry results can be explained if we assume that the adsorbed layer has two effects. First, it increases the anisotropy of the potential affecting the electrons’ spins at the interface due to charge transfer between the substrate and the adsorbed DNA and thereby increases the magnetic moment measured at room temperature. Second, it has, by itself, diamagnetic properties. At lower coverage, the anisotropy effect matters more than the DNA film’s diamagnetism, and the magnetic moment increases relative to the bare surface, but for the higher coverage condition, the contribution of the diamagnetism matters more and decreases the magnetic moment back toward that of the bare surface (shown in Figure S6).

To verify the role of the charge transfer, the contact potential difference was measured by cyclic voltammetry for fully matched DNA adsorbed on a surface that is not magnetized, surface-magnetized North, and surface-magnetized South. The measurements were repeated four times, and in each case the sample was collected in the order of no magnet, North, and South poles pointing toward the adsorbed molecules. The contact potential results are shown in Table 3. When comparing each trial, the data are consistent with the contact potential becoming more negative with the higher coverage surface (South pole). The difference between each trial may arise from slight differences in the concentration of DNA in solution. Although these differences are quite small, the data are consistent with the contact potential becoming more negative with the higher coverage surface (South). This finding is consistent with more charge moving from the surface to the adsorbed layer for the North pole aligned toward the molecules, relative to the South pole. This interpretation is consistent with former studies indicating that the amount of charge injected into a layer adsorbed on a ferromagnetic surface depends on the direction of the magnetization of the layer for chiral molecules.^5^ These findings show that the difference observed in the coverage correlates with differences in the total charge and the magnetic properties of the DNA layer.

**Table 3:** Contact Potential, in Units of V, on the Ni/Au Films Is Reported for the Full-Matched DNA in Solution

trial	No magnet	North	South
1st	–0.1574	–0.1547	–0.1595
2nd	–0.1589	–0.1588	–0.1628
3rd	–0.1626	–0.1605	–0.1650
4th	–0.1609	–0.1600	–0.1642
average	–0.1560 ± 0.0022	–0.1585 ± 0.0026	–0.1629 ± 0.0024

## Conclusions

This study reveals that the adsorption of OG-damaged DNA on ferromagnetic substrates is spin-selective and depends on the location of the damage in the DNA helix. For three of the different types of duplexes studied, the adsorption rate was faster and the coverage was higher on a South-magnetized FM surface than on a North-magnetized surface. For the distal OG-damaged DNA, the differences were less significant (similar rates, but somewhat higher coverages in the fluorescence experiments). The dependence on the magnetic field was rationalized in terms of a coupling between charge polarization and spin polarization in the DNA duplex, and the dependence of the adsorption asymmetry on the position of the OG damage was rationalized by differences in the charge polarization through the molecular monolayers. Although these studies were performed on an artificial system, they suggest a new contribution to the interactions between chiral molecules; e.g., when a protein (or enzyme) interacts with DNA, their charge polarizations are accompanied by spin polarizations that can change their interaction strength. Moreover, the strength of the interaction will be sensitive to the damage to DNA and its location.

## Supporting Information Available

The file contains XPS spectra of the DNA-adsorbed Ni/Au substrates, cyclic voltammograms and corresponding mass changes in the quartz crystal microbalance measurement, CD spectra of the full-match and OG DNA before and after annealing, CD spectra of a 20 bp full-match DNA and its adsorption kinetics on the Ni/Au substrate, adsorption of full-match 40 bp DNA on the Au electrode monitored by QCM, and SQUID results of the full-match and central OG DNA adsorbed on Ni/Au (PDF)

## References

[ref1] Neeley W. L.; Essigmann J. M. Mechanisms of Formation, Genotoxicity, and Mutation of Guanine Oxidation Products. Chem. Res. Toxicol. 2006, 19, 491–505. 10.1021/tx0600043. 16608160

[ref2] David S. S.; O’Shea V. L.; Kundu S. Base-Excision Repair of Oxidative DNA Damage. Nature 2007, 447, 941–950. 10.1038/nature05978. 17581577 PMC2896554

[ref3] Mishra S.; Poonia V. S.; Fontanesi C.; Naaman R.; Fleming A. M.; Burrows C. J. Effect of Oxidative Damage on Charge and Spin Transport in DNA. J. Am. Chem. Soc. 2019, 141, 123–126. 10.1021/jacs.8b12014. 30541275

[ref4] Naaman R.; Waldeck D. H.; Paltiel Y. Chiral Molecules-Ferromagnetic Interfaces, an Approach towards Spin Controlled Interactions. Appl. Phys. Lett. 2019, 115, 133701 10.1063/1.5125034.

[ref5] Ghosh S.; Mishra S.; Avigad E.; Bloom B. P.; Baczewski L. T.; Yochelis S.; Paltiel Y.; Naaman R.; Waldeck D. H. Effect of Chiral Molecules on the Electron’s Spin Wavefunction at Interfaces. J. Phys. Chem. Lett. 2020, 11, 1550–1557. 10.1021/acs.jpclett.9b03487. 32013436 PMC7307953

[ref6] Zhu Q.; Kapon Y.; Fleming A. M.; Mishra S.; Santra K.; Tassinari F.; Cohen S. R.; Das T. K.; Sang Y.; Bhowmick D. K.; et al. The role of electrons’ spin in DNA oxidative damage recognition. Cell Rep. Phys. Sci. 2022, 3, 101157 10.1016/j.xcrp.2022.101157.

[ref7] Lu Y.; Bloom B. P.; Qian S.; Waldeck D. H. Enantiospecificity of Cysteine Adsorption on a Ferromagnetic Surface: Is It Kinetically or Thermodynamically Controlled?. J. Phys. Chem. Lett. 2021, 12, 7854–7858. 10.1021/acs.jpclett.1c02087. 34380316

[ref8] Banerjee-Ghosh K.; Dor O.; Ben; Tassinari F.; Capua E.; Yochelis S.; Capua A.; Yang S. H.; Parkin S. S. P.; Sarkar S.; Kronik L.; et al. Separation of Enantiomers by Their Enantiospecific Interaction with Achiral Magnetic Substrates. Science 2018, 360, 1331–1334. 10.1126/science.aar4265. 29748324

[ref9] Johnson M. Bipolar Spin Switch. Science 1993, 260, 320–323. 10.1126/science.260.5106.320. 17838246

[ref10] Johnson M. Spin Polarization of Gold Films via Transported. J. Appl. Phys. 1994, 75, 6714–6719. 10.1063/1.356848.

[ref11] Santra K.; Zhang Q.; Tassinari F.; Naaman R. Electric-Field-Enhanced Adsorption of Chiral Molecules on Ferromagnetic Substrates. J. Phys. Chem. B 2019, 123, 9443–9448. 10.1021/acs.jpcb.9b07987. 31609607

[ref12] Bloom B. P.; Graff B. M.; Ghosh S.; Beratan D. N.; Waldeck D. H. Chirality Control of Electron Transfer in Quantum Dot Assemblies. J. Am. Chem. Soc. 2017, 139, 9038–9043. 10.1021/jacs.7b04639. 28609095

[ref13] Abendroth J. M.; Nakatsuka N.; Andrews A. M.; Ye M.; Kim D.; Fullerton E. E.; Weiss P. S. ACS Nano 2017, 11, 7516–7526. 10.1021/acsnano.7b04165. 28672111

[ref14] Wei J. J.; Schafmeister C.; Bird G.; Paul A.; Naaman R.; Waldeck D. H. Molecular Chirality and Charge Transfer through Self-Assembled Scaffold Monolayers. J. Phys. Chem. B 2006, 110, 1301–1308. 10.1021/jp055145c. 16471678

[ref15] Kumar A.; Capua E.; Kesharwani M. K.; Martin J. M. L.; Sitbon E.; Waldeck D. H.; Naaman R. Chirality-Induced Spin Polarization Places Symmetry Constraints on Biomolecular Interactions. Proc. Natl. Acad. Sci. U.S.A. 2017, 114, 2474–2478. 10.1073/pnas.1611467114. 28228525 PMC5347616

[ref16] Markus T. Z.; Daube S. S.; Naaman R.; Fleming A. M.; Muller J. G.; Burrows C. J. Electronic Structure of DNA-Unique Properties of 8-Oxoguanosine. J. Am. Chem. Soc. 2009, 131, 89–95. 10.1021/ja804177j. 19128174

