# Supplementary material for: Spin Selectivity Damage Dependence of Adsorption of dsDNA on Ferromagnets
Source: J Phys Chem B. 2023 Mar 8;127(11):2344–50. doi: 10.1021/acs.jpcb.2c08820 (PMC10041612; doi:10.1021/acs.jpcb.2c08820)
Supplement: Supplementary file 1 — jp2c08820_si_001.pdf [file jp2c08820_si_001.pdf]

Supporting Information

Spin Selectivity Damage Dependence of Adsorption of dsDNA on Ferromagnets

Kakali Santra[+1], *Yiyang Lu*[+2], *David H. Waldeck*[*2], and *Ron Naaman*[*1]

[1]Department of Chemical and Biological Physics, Weizmann Institute, Rehovot 76100, Israel.

[2] Chemistry Department, University of Pittsburgh; Pittsburgh, Pennsylvania 15260, United States.

Contents

Supporting Figures 2

Figure S1: XPS spectra of the adsorbed full-match DNA on Ni/ Au (100/ 5 nm) substrate magnetized with the North pole oriented either Up or Down. Panel (A) shows the elemental S 2p signal, and panel (B) shows the elemental N 1s signal where the intensity is plotted as a function of binding energies (eV). 2

Table S1: Elemental percentage calculated from XPS measurements 2

Figure S2: Cyclic voltammogram (A) and corresponding mass change (B) for the electrode in contact with a 0.3 µM DNA solution in 0.4 M pH 7.2 phosphate buffer. 3

Figure S3: CD (A) and absorption (B) spectra of the full match ssDNA and dsDNA helices. CD (C) and absorption (D) spectra of the hybridized dsDNA molecules (distal, central and proximal OG and full match). 4

Figure S4: (A). CD spectrum of 20-bp full match DNA sequence. (B). Spin-dependent adsorption of the double helix 20 base pair DNA molecules on a Ni/ Au coated ferromagnetic (FM) film by confocal microscopy 5

Figure S5: Results for the spin-dependent adsorption of the 0.5 µM full match DNA molecules on a 100 nm Au coated film by quartz crystal microbalance. 5

Figure S6: SQUID results of (A) full match DNA and (B) central OG DNA adsorbed on Ni/Au subsrate when the substrate was magnetized either with the magnet North or South pole up direction. The bare Ni/Au substrate was measured as a reference. Measurements were conducted at 300 K. 6

Supporting Figures

XPS Measurements:

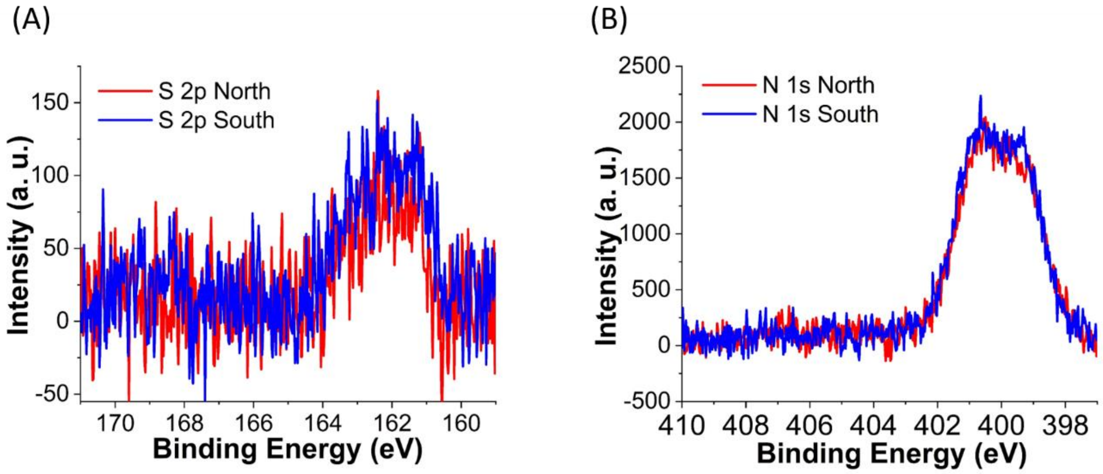

Figure S1: XPS spectra of the adsorbed full-match DNA on Ni/ Au (100/ 5 nm) substrate magnetized with the North pole oriented either Up or Down. Panel (A) shows the elemental S 2p signal, and panel (B) shows the elemental N 1s signal where the intensity is plotted as a function of binding energies (eV).

Table S1: Elemental percentage calculated from XPS measurements

elements	North, point1	North, point 2	South, point 1	South, point 2
N 1s/ Au 4f	7.38/28.47 =0.26	7.3/28.98 =0.25	6.97/28.87 =0.24	7.33/28.07 =0.26
S 2p/ Au 4f	0.21/28.47 =0.007	0.27/28.98 =0.009	0.34/28.87 = 0.11	0.32/28.07 =0.11

QCM Measurements:

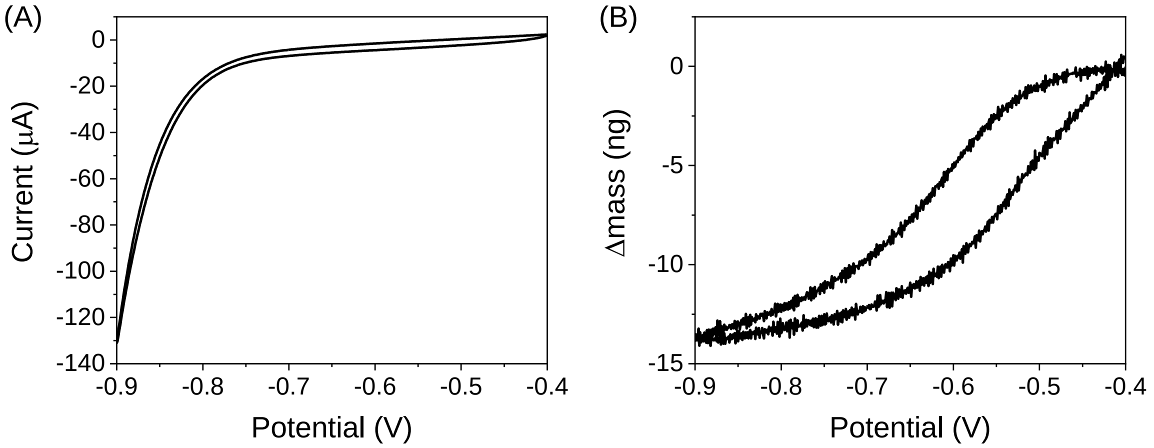

Figure S2: Cyclic voltammogram (A) and corresponding mass change (B) for the electrode in contact with a 0.3 μM DNA solution in 0.4 M pH 7.2 phosphate buffer.

CD Spectra:

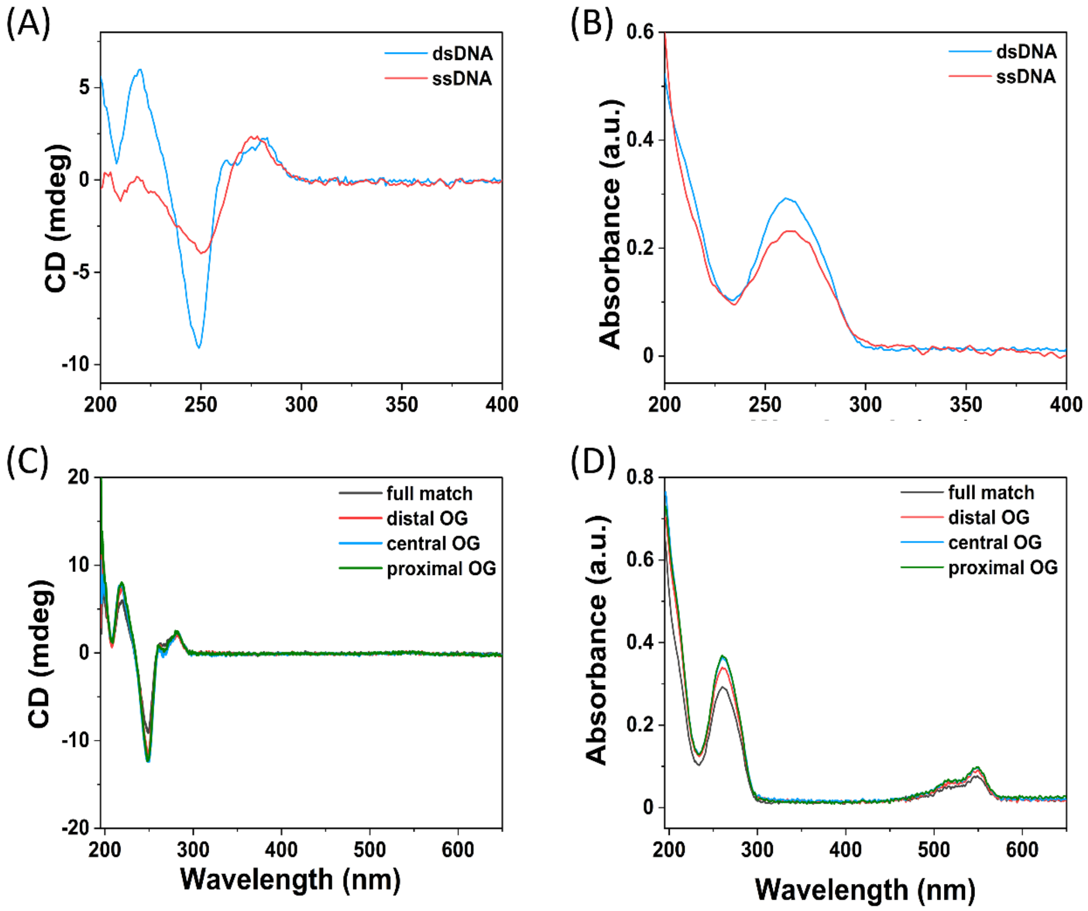

Figure S3: CD (A) and absorption (B) spectra of the full match ssDNA and dsDNA helices. CD (C) and absorption (D) spectra of the hybridized dsDNA molecules (distal, central and proximal OG and full match).

CD spectra and adsorption kinetics of the full match 20 base pair DNA:

Sequence:

5' GAC CAC AGA TTC AAA CAT GC - *Thiol-Modifier-C3 S-S* **3'**

3' *Cy3* – CTG GTG TCT AAG TTT GTA CG **5'**

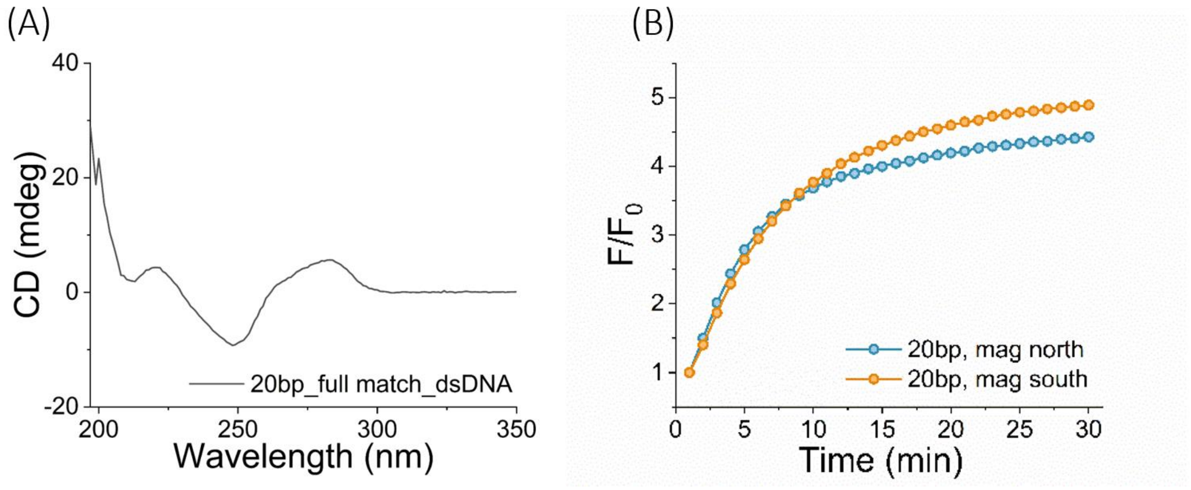

Figure S4: (A). CD spectrum of 20-bp full match DNA sequence. (B). Spin-dependent adsorption of the double helix 20 base pair DNA molecules on a Ni/ Au coated ferromagnetic (FM) film by confocal microscopy

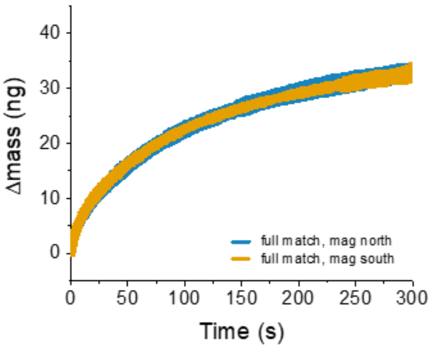

Figure S5: Results for the spin-dependent adsorption of the 0.5 μM full match DNA molecules on a 100 nm Au coated film by quartz crystal microbalance.

SQUID measurements

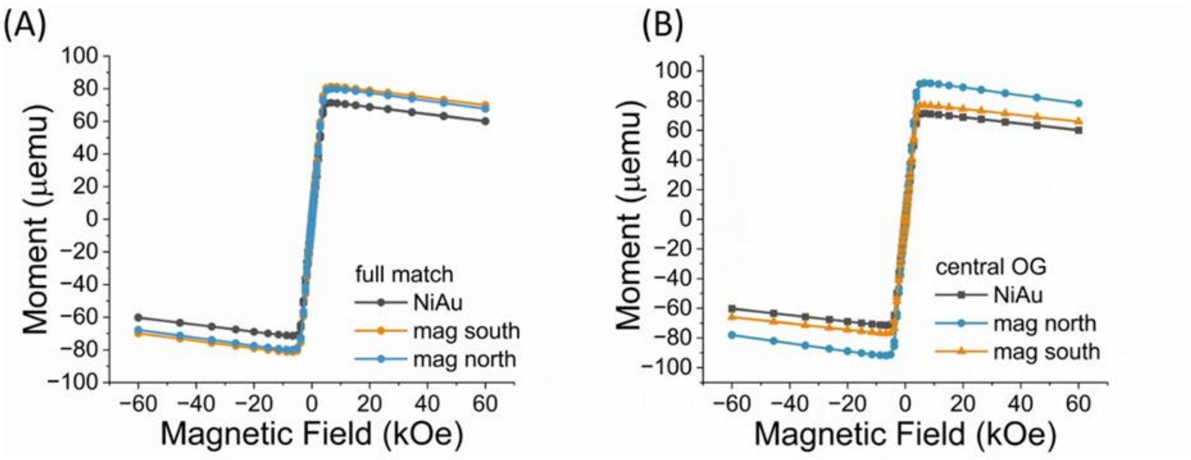

Figure S6: SQUID results of (A) full match DNA and (B) central OG DNA adsorbed on Ni/Au subsrate when the substrate was magnetized either with the magnet North or South pole up direction. The bare Ni/Au substrate was measured as a reference. Measurements were conducted at 300 K.

Error Calculation:

The Propagation of Error Calculation Formula Used for Table 2:

$$\Delta(F/M) = (F/M) \cdot \sqrt{\left(\frac{\Delta F}{F}\right)^2 + \left(\frac{\Delta M}{M}\right)^2}$$

ignoring the cross term $\left(\frac{-2\Delta F.\Delta M}{F.M}\right)$; i.e., assuming that the errors are not correlated.
